# Self‐directed speech and attention deficit hyperactive disorder‐like behaviours

**DOI:** 10.1111/bjop.70027

**Published:** 2025-09-19

**Authors:** Ellie Benfield, Geoff G. Cole

**Affiliations:** ^1^ Centre for Brain Science University of Essex Colchester UK

**Keywords:** ADHD, camouflaging, inner speech, self‐talking

## Abstract

One of the behaviours associated with Attention Deficit Hyperactive Disorder (ADHD) is talking in a manner considered to be socially inappropriate. It follows, therefore, that self‐directed speech, including inner dialogue, will be particularly prevalent among adults who exhibit traits typical of ADHD. In three experiments, we tested this prediction. Participants completed the ASRS‐v1.1 together with either the *Self‐Talk Scale* (Experiment 1; *N* = 198) or the *Varieties of Inner Speech Questionnaire* (Experiment 2; *N* = 198). Results from both experiments revealed that participants with behaviours typical of an ADHD diagnosis reported significantly more self‐directed speech than those whose behaviours were not typical of ADHD. A third experiment (*N* = 198) replicated these findings and also found that the effect does not distinguish between overt and covert speech. Overall, these data suggest that self‐talking is more prevalent in individuals with relatively high levels of ADHD traits. We speculate that talking to oneself may represent a useful displacement activity or acts as a camouflage technique for those with the condition.

## BACKGROUND

Attention Deficit Hyperactive Disorder (ADHD) is a relatively common neurodevelopmental disorder characterized by inattention, impulsivity, and hyperactivity (Lahey et al., 1988). The condition affects approximately 4.4% of the adult population and occurs with varying degrees of severity (Beau‐Lejdstrom et al., 2016). Although it is often associated with children, as many as 50% of individuals diagnosed with ADHD in childhood still have the condition in adulthood (Okie, 2006). Misdiagnosis is common as symptoms can mimic those of other disorders. For example, overstimulation is prevalent in many neurodevelopmental conditions such as autism spectrum disorder (ASD) and generalized anxiety disorder (Fusar‐Poli et al., 2022; Magnin & Maurs, 2017). The difficult, and often expensive, process of obtaining an ADHD diagnosis (Loh et al., 2022) regularly results in individuals being deterred from seeking an official examination and preferring to employ their own coping strategies (Young, 2005). As Barra et al. (2021) observed, individuals who show high ADHD symptomatology frequently employ maladaptive stress coping strategies leading to, for instance, increased social withdrawal. This may in turn lead to other longer‐term issues, such as developing depression, anxiety, social isolation and low self‐esteem (Merrell & Gimpel, 2014). Furthermore, in a comprehensive review of functional impairments associated with ADHD, Kosheleff et al. (2023) reported greater frequency of problems relating to work and economic security (Murphy & Barkley, 1996), forming and maintaining romantic relationships (Michielsen et al., 2015), avoiding personal injury (Merrill et al., 2009) and substance use (Molina et al., 2018), among many others.

As noted, individuals with ADHD often have difficulty with everyday social skills, and a large array of symptoms is considered to be socially unacceptable. For example, interrupting others speaking, excessive fidgeting and being inattentive to conversations is common. The latter is often due to the fact that individuals with ADHD can be easily distracted or simply lack interest in the current situation (Sroubek et al., 2013). This often leaves people with ADHD being left out or not accepted by their peers (Bellanti & Bierman, 2000). In children, inappropriate play behaviours have also been linked to peer rejection (Harkins et al., 2022). Being inattentive during group activities, violating rules in games, being controlling and easily frustrated are also common (Guevremont & Dumas, 1994). Richards et al. (2002) also found that high ADHD‐symptom college students experienced relatively more anger while driving and expressed this in particularly hostile and aggressive ways.

One of the behaviours associated with ADHD, and often deemed socially inappropriate is excessive talking. This is reflected in the ASRS‐v1.1 (Kessler et al., 2005), one of the most commonly used tools used to assess the phenomenon. For example, one item asks, ‘When you're in a conversation, how often do you find yourself finishing the sentences of the people you are talking to, before they can finish them themselves?’ Another asks, ‘How often do you find yourself talking too much when you are in social situations?’ Given that various types of inappropriate talking is one of the traits, one can reasonably predict that ADHD will be associated with greater prevalence of *self‐directed speech*.

As the name suggests, self‐directed speech is the behaviour in which a person produces dialogue directed solely towards themselves. This can comprise both internal private monologue, such as imagined conversations (Alderson‐Day et al., 2018; see Alderson‐Day & Fernyhough, 2015, for review), or overt speech (Berk, 1992). Given the importance of language for child development, particularly in the context of Vygotsky's work (Vygotsky, 1934/1987), the phenomenon has been examined extensively in children. For example, use of private speech has been shown to occur in infants aged 23–25 months (Furrow, 1984) before increasing with age and peaking at between 4 and 5 years (Winsler et al., 2003). It is then typically replaced gradually by partially internalized mutterings and whispers between 5 and 10 years (see Bivens & Berk, 1990; Manning & White, 1990). Although particularly prevalent in younger children, self‐directed speech continues to be used in adolescence and adulthood. For instance, Winsler and Naglieri (2003) observed overt self‐talking during a cognitive task in 10%–30% of individuals aged 11–17.

With respect to use and function, self‐directed speech has been associated with a large range of behaviours and tasks. These appear to benefit from the self‐regulatory, reflective and management aspects of the phenomenon. Such behaviours and tasks include those concerned with, for example, problem‐solving, decision‐making, emotional processing, working memory, resisting temptation, attentional focus, mind‐wandering, imagination and creativity (see Morin & Racy, 2022, for review). In children, one experimental paradigm has been to observe the degree (or absence) of self‐directed speech, and the type employed, while tasks of varying difficulty are undertaken. For example, Fernyhough and Fradley (2005) videotaped children aged 5–6 years performing four progressively difficult starting positions of the *Tower of London* task. Results revealed differences in the amount of self‐talk dependent on difficulty. When the task was relatively easy, self‐directed speech was infrequent. This was also the case when the task was difficult. In contrast, an intermediate level of difficulty resulted in the most frequent self‐directed speech. Following Behrend et al. (1989), Fernyhough and Fradley suggested that when the task is too simple, self‐talk will be unnecessary, because the regulatory processes provided by such talk will already have been internalized. When the task is too difficult, self‐talk will be ineffective, resulting either in other means of regulation or failure on the task.

A similar experimental rationale has been applied in older children and adults. That is, tasks of varying difficulty are employed while varying levels of self‐directed speech occur. In contrast to experiments with younger children, these paradigms will often manipulate whether self‐talk occurs at all, via articulatory suppression. For example, Lidstone et al. (2010) compared *Tower of London* performance in children aged 7–10 years who undertook either the standard version of the task, during articulatory suppression, or during foot‐tapping. The latter condition effectively acts as a non‐verbal‐task control. Lidstone et al. found that correct performance was impaired during articulatory suppression, thus supporting a causal role for self‐directed speech. Interestingly, the authors only found this effect when participants were asked to *mentally plan* moves before they were performed. This concurs with the notion, proposed by Vygotsky, that self‐directed speech is particularly useful when planning. Another paradigm, typically employed in adults, is to require participants to use either positive or negative self‐talk while performing a difficult task. For example, during a dart throwing task, Van Raalte et al. (1995) asked undergraduate students to either say ‘You can do it’ or ‘You cannot do it’ before each throw. Results showed that the former led to increased performance. This concurs with typical findings seen in the applied sport psychology literature (e.g., Gould et al., 1989).

Very little work has, however, examined self‐directed speech in adults with ADHD. In a recent review of inner speech and neurodiversity, Alderson‐Day and Pearson (2023) stated that ‘almost no studies have explored self‐directed speech in adults with ADHD, dyslexia, or a history of language difficulties. The role of this process in diverse examples of cognition is therefore at best underexplored, and at worst assumed’ (p. 197). The aim of the present work therefore was to examine whether self‐directed speech is more common within adults who exhibit relatively high levels of ADHD‐like behaviours compared to those individuals who do not. We predicted this to be the case based on the rationale that ADHD is known to be associated with excessive talking, which is often deemed to be socially inappropriate. One might therefore expect this behaviour to also manifest itself as greater frequency of self‐directed speech. We defined individuals with ADHD‐like behaviours via scores on two commonly employed inventories.

## EXPERIMENT 1

In Experiment 1, participants completed the ASRS‐v1.1 together with the *Self‐Talk Scale* (STS). Brinthaupt et al. (2009) developed the STS to assess the frequency of self‐talking in nonclinical adult populations. They found the construct to be closely related to four factors, namely *social assessment*, *self‐reinforcement*, *self‐criticism* and *self‐management*. In addition to the initial validation work of Brinthaupt et al., the scale was further assessed by Brinthaupt and Kang (2014) using the Rasch model (Rasch, 1980) of test development, an approach designed to circumvent some of the limitations that arise from traditional measurement work based on classical test theory (e.g., Allen & Yen, 2001). Furthermore, Uttl et al. (2011) found that the STS performed well compared to three other commonly used self‐talk scales. It is also the most commonly cited self‐directed speech scale, despite not being the oldest. Similarly, the *ADHD Self Report Scale* (ASRS‐v1.1, Kessler et al., 2005) is a commonly used questionnaire. The 18‐item scale, developed in conjunction with the *World Health Organization*, asks respondents to consider the degree to which they exhibit a number of behaviours associated with attention, impulsivity and hyperactivity. In a review and assessment of 14 similar inventories, all designed to identify those with ADHD‐typical behaviours, Taylor et al. (2011) found that the ASRS‐v1.1 generated the highest overall diagnostic accuracy. This was true for both the full 18‐item version and the 6‐item ‘screener’.

### Method

#### Participants

Two‐hundred and seven participants were recruited via the *Prolific* participation platform. They were screened to be male or female, have English as their first language, be aged between 18 and 75 and be UK or US citizens. In order to be included in the formal analysis, participants were required to answer all questions, give informed consent and correctly respond to an attention/bot check question. This stated, ‘This question is a bot/attention check. Please press the Number 6’. Six participants failed this check and were not included in further analyses. As per our preregistration, we then took the first 198 participants (ensuring exact counterbalancing of the two questionnaires, i.e., the first 99 from each) who completed the study according to the above inclusion criteria and these were included in the analysis. Of these participants, 96 indicated their sex as female and 98 as male. Four participants did not indicate their sex. Ages ranged from 18 to 70 (M = 40.8, SD = 13.4). Three participants did not provide their age. They were paid £1.10 (or US equivalent) for taking part.

#### Materials, procedure and analysis

Participants were first asked to indicate their sex and age (or respond ‘Prefer not to say’). They then completed the 18‐item ASRS‐v1.1 and the 16‐item STS. The presentation order of these was counterbalanced such that (of those included in the formal analyses) 99 participants completed the ASRS‐v1.1 first and 99 the STS. The latter asks people to respond to each item on a 5‐point Likert scale ranging from ‘Never’ to ‘Very Often’. Because (weighted) scores on the 6‐item screener of the ASRS‐v1.1 have strong concordance with a clinical diagnosis (Kessler et al., 2007) we defined participants as having ADHD‐like traits if they scored 2 or above (on a 0–4 scale) on Items 1–3 and 3 or above on items 4–6, as recommended by Kessler et al. (2007). The method and analyses were preregistered at https://osf.io/tev6r and an update at https://osf.io/nxqft. Experiments 2 and 3 were not pre‐registered. The present experiments were approved by the University of Essex Psychology Research Ethics Committee (Reference number: ETH2223‐1070) entitled ‘Self‐talking and ADHD’.

The critical effect we are examining in the present three experiments is self‐directed speech in those with ADHD‐like traits and those without. In terms of power and effect size, our sample size of 198 participants (employed in the formal analyses) is large enough to detect a small to medium effect (i.e., Cohen's *d* = 0.4), using an alpha of .05 and 80% power (using G*Power). To put another way, our sample size generates a 93.7% probability of detecting a medium effect. Note that the *American Psychological Association* suggests that researchers can alternatively assume a medium effect corresponds to a Cohen's *d* of 0.95 (*η* ^2^ = 0.06), rather than 0.5. This is based on a sample of experimental psychology research findings (as opposed to, e.g., social psychology or applied psychology) that Schäfer and Schwarz (2019) examined (see also Correll et al., 2020) showing that Cohen had underestimated the median size of experimental research results. If we were to adopt this suggestion, our power to detect a medium effect increases to over 99%, and our sample sizes have the ability to detect a very small effect.

### Results and discussion

**FIGURE 1:**
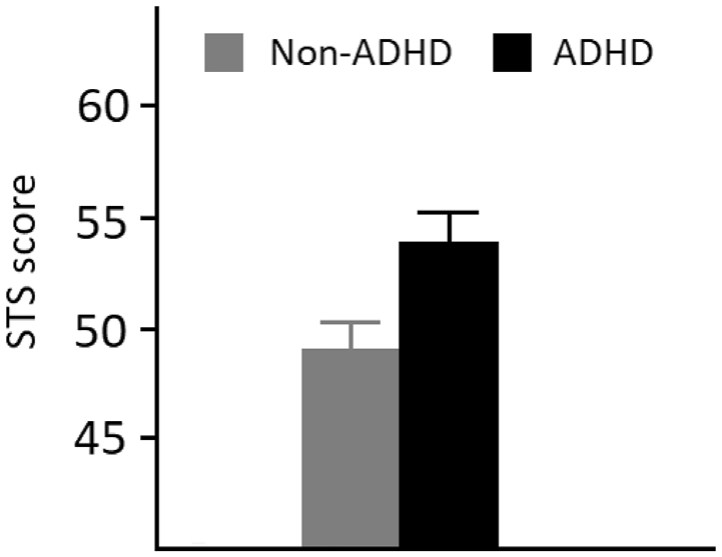
Means and standard errors from Experiment 1.

The two measures used generated good internal reliability (STS, *α* = .93; ASRS‐v1.1, *α* = .9). Overall mean ASRS‐v1.1 score was 29.1 (SD = 12.2) with 56 participants reaching the criterion for ADHD‐like behaviours classification. Figure 1 shows the mean STS score for the two conditions. The central analysis compared the mean STS score for participants who reached the ASRS‐v1.1 definition for ADHD and those who did not. An independent samples *t*‐test revealed this difference to be significant, *t*(196) = 2.4, *p* = .02, Cohen's *d* = 0.37. We also found a significant correlation between STS score and ASRS‐v1.1 score, *r*(196) = .436, *p* < .001. These results show that individuals with ADHD‐like behaviours report relatively greater self‐talking. Additionally, an unplanned analysis examined any differences between ADHD and non‐ADHD individuals with respect to the four factors that Brinthaupt et al. (2009) identified in the STS, that is, *social assessment, self‐criticism, self‐management* and *self‐reinforcement*. We found a significant difference in all factors: *t*(196) = 3.0, *p* < .01; *t*(196) = 2.8, *p* < .01; *t*(196) = 2.4, *p* < .02, respectively, with the sole exception of *self‐reinforcement* (*t* = 0.49, *p* = .63).

Our original pre‐registration stated that we would define ADHD‐like traits by performing a median split on the ASRS‐v1.1 scores. As noted in the Method section above, we updated this registration stating that we would instead define ADHD using the ASRS‐v1.1 screener (i.e., the first six items of the scale). Appendix S1, which can be found at https://osf.io/kdvqe/files/osfstorage, presents the results from all three of the present experiments using the median split definition. These results show that the basic self‐talking effect we report in the present manuscript is larger when median split analyses are performed.

## EXPERIMENT 2

Brinthaupt et al. (2009) referred to *self‐talk* as the behaviour in which either private inner thinking or external speech is generated. The phenomenon can, however, be distinguished between these two. This is reflected in the variety of terms used, such as *inner monologue*, *auditory imagery*, *private speech*, *self‐talking, internal speech*, *verbal thoughts* and *self‐statements*. As the name suggests, the *Varieties of Inner Speech Questionnaire* (VISQ; Alderson‐Day et al., 2018) was developed to examine the different types or functions of *inner speech* only. Five types were described: dialogue, as an evaluative/motivational device; when other people's voices are imagined; as abbreviated sentences that retain meaning and positive/regulatory. As reported by Alderson‐Day et al. in their initial article, the VISQ demonstrates good internal and test–retest reliability. It is also associated with measures of anxiety, depression and auditory (but not visual) hallucinations (McCarthy‐Jones & Fernyhough, 2011). The scale has additionally been used to examine the relationship between inner speech and psychosis (de Sousa et al., 2016). In Experiment 2, (new) participants completed the ASRS‐v1.1 together with the revised version (i.e., Alderson‐Day et al., 2018) of the VISQ. The aim was to examine whether purely inner speech is also particularly prevalent among people with ADHD‐like symptoms.

### Method

All aspects of the method were identical to Experiment 1 with the exception that 219 participants initially undertook the experiment. The same exclusion criteria were also applied and again six people failed the attention/bot check question. The first 198 participants were included in the formal analysis (the first 99 from the two counterbalanced blocks). Ages from these participants ranged from 18 to 75 and the mean was 39.9 years (SD = 12.9). Ninety participants indicated they were male and 102 female. Sex and age data from six participants were not provided.

### Results and discussion

**FIGURE 2:**
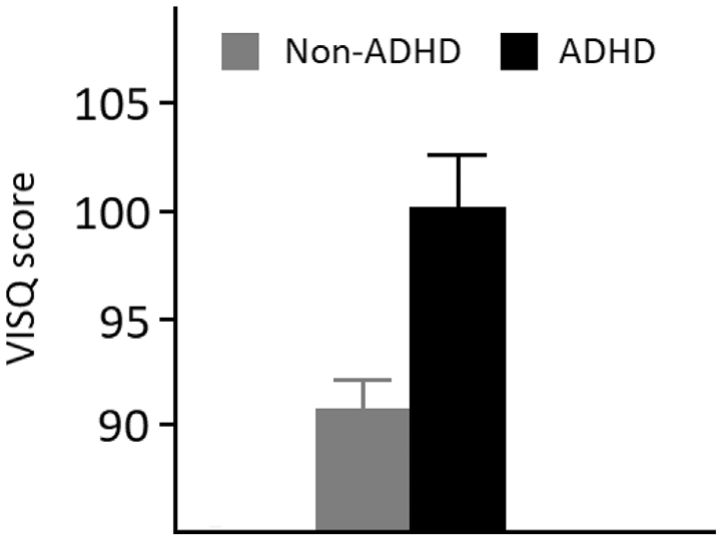
Means and standard errors from Experiment 2.

The ASRS‐v1.1 generated good reliability (*α* = .93) as did the VISQ (*α* = .83). Overall mean ASRS‐v1.1 score was again 29.1 (14.1) and 55 participants reached the threshold for ADHD‐like traits classification. Figure 2 shows the mean STS score for the two conditions. An independent sample *t*‐test showed this difference to be significant, *t*(196) = 3.1, *p* = .002, Cohen's *d* = 0.49. There was also a significant positive correlation between ASRS‐v1.1 scores and VISQ scores, *r*(196) = .504, *p* < .001. These data thus show that individuals with ADHD‐like behaviours report relatively greater inner‐speaking relative to non‐ADHD‐like individuals. Overall, these results support the findings from Experiment 1. As with Experiment 1, we again undertook an unplanned analysis assessing any differences between ADHD and non‐ADHD individuals with respect to the scale's subfactors. These results showed that whilst *dialogue* [*t*(196) = 2.4, *p* < .02], *evaluative*/*motivational* [*t*(196) = 4.1, *p* < .001], and *other voices imagined* [*t*(196) = 2.3, *p* < .03] showed a significant difference, *abbreviated sentences* [*t*(196) = 0.98, *p* > .32] and *positive/regulatory* [*t*(196) = 0.544, *p* > .58] did not.

## EXPERIMENT 3

The designs and data from Experiments 1 and 2 do not allow us to assess any differences between covert inner speech and overt self‐talking. This is because the VISQ measures inner speech only and the STS measures *both* inner and overt speech. In Experiment 3, we directly examined whether individuals with ADHD‐like traits differ from individuals with non‐ADHD‐like traits in the degree to which they employ covert and overt speech. Participants again completed the ASRS‐v1.1 and, in two additional questions, were asked to indicate the degree to which they employ inner and overt self‐talking. They were also asked two open‐ended questions in which they were invited to briefly describe the situations in which they employ these.

### Method

Participants were recruited via social media and the University of Essex participant recruitment platform. Three‐hundred and thirty participants initiated the study. After the same exclusion criteria as described previously was applied, 198 participants completed the procedure. Of these participants, 149 indicated they were female and 49 male. Age ranged from 18 to 83, with the mean age being 42.0 (SD = 15.5). After the demographic questions were answered, the 18‐item ASRS‐v1.1 was administered (together with the bot/attention check item) followed by the two questions of central interest concerning self‐talking. These stated, *How often do you talk to yourself out loud?* and *How often do you talk to yourself in your head?* As with the other items (on the ASRS‐v1.1), participants indicated *Never, Rarely, Sometimes, Often* or *Very Often*. They were also asked eight other questions concerning any ADHD‐like symptoms. These were not however subject to any analysis. They were finally asked two open‐ended question: *In about 2–3 sentences, can you describe the circumstances in which you talk to yourself out loud?* and *In about 2‐3 sentences, can you describe the circumstances in which you talk to yourself in your head?* We effectively manipulated ADHD‐like traits (ADHD, non‐ADHD) and type of speech (overt, covert) in a 2 × 2 mixed design.

### Results and discussion

**FIGURE 3:**
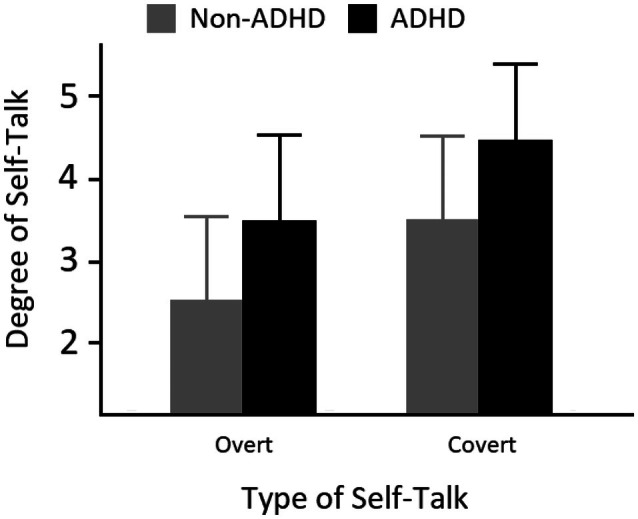
Results from Experiment 3. Standard deviations are also shown.

The ASRS‐v1.1 again generated good reliability (*α* = .94). Overall mean ASRS‐v1.1 score was 33.7 (SD = 14.0) and 76 participants reached the threshold for ADHD classification. Recall that the central issue concerns whether ADHD‐like and non‐ADHD‐like participants differ in the degree to which they self‐talk overtly or covertly. Figure 3 shows the mean responses on the two self‐talk questions for those who reached the threshold for ADHD and those who did not. A 2 × 2 mixed ANOVA revealed a significant main effect of ADHD‐like behaviours, *F*(1, 196) = 30.0, *p* < .001, *d* = 0.58, and a significant main effect of speech type, *F*(1, 196) = 134.0, *p* < .001, *d* = 0.79. There was, however, no significant interaction, *F*(1, 196) = 0.42, *p* > .51, Cohen's *d* = 0.07. The correlation between ASRS‐v1.1 scores and scores on the two covert/overt speech questions was significant, *r*(196) = .52, *p* < .001. Overall, the data show that although self‐directed speech is more prevalent in individuals with ADHD‐like traits (thus replicating Experiments 1 and 2), this effect does not distinguish between its two varieties (i.e., overt and covert speech).

#### Open‐ended questions

**FIGURE 4:**
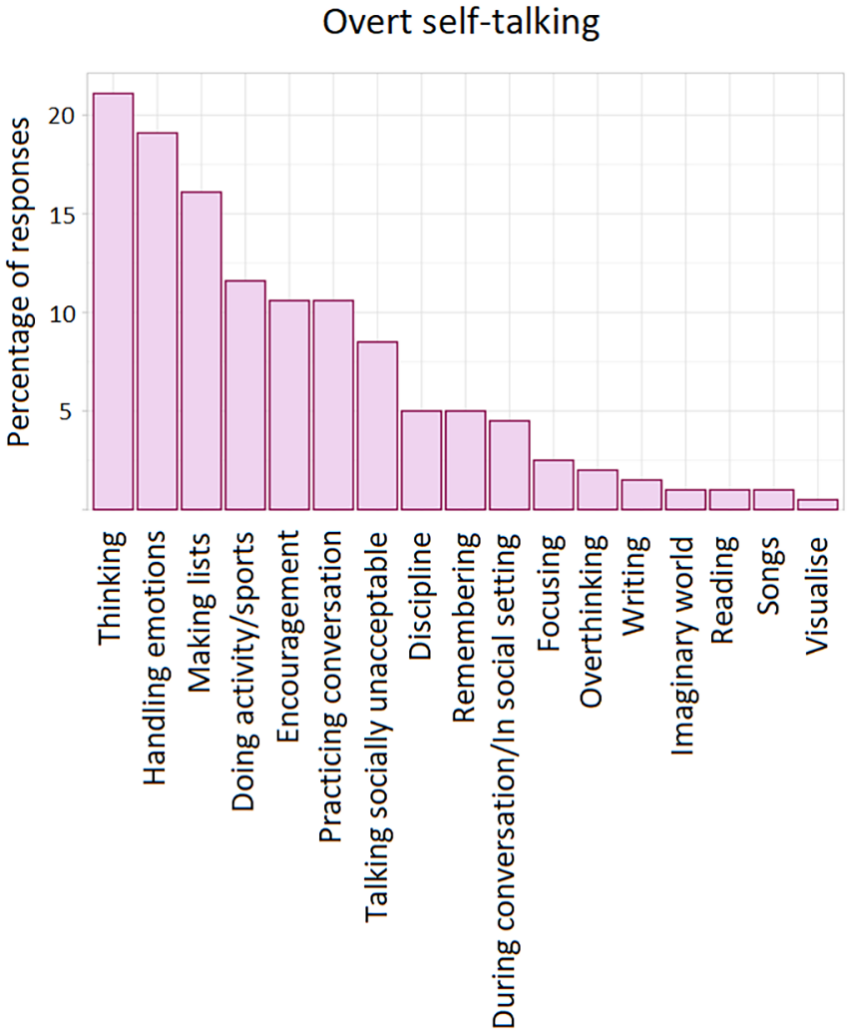
Scenarios associated with overt self‐talking.

**FIGURE 5:**
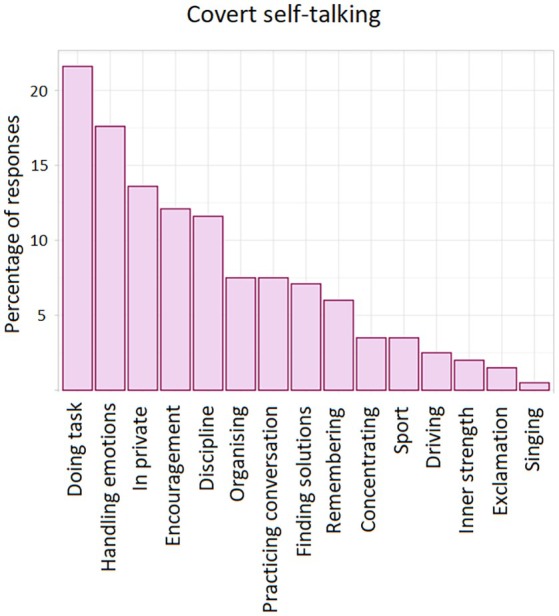
Scenarios associated with covert self‐talking.

One hundred and fifty‐seven people (out of 198) answered at least one of the two questions. The mean number of words provided by participants who wrote at least one word was 13.6. We generated a number of themes from these responses. These are shown in Figures 4 and 5. As the analysis shows, common situations (or reasons) in which self‐talking occurs, both overt and covert, are to assist in the handling of emotions, for undertaking tasks, and for self‐motivation. We also uploaded all participant responses for the two open‐ended questions to the AI software *Google Notebook LM*. A small number of peer‐reviewed articles have now assessed a range of AI tools for their ability to adequately undertake a thematic analysis. Bennis and Mouwafaq (2025), for example, found that *Google Notebook LM* recorded ‘strong performance’. The results of our AI analysis (i.e., the raw text output) are presented in the Appendix S1. We find that this shows good consistency with the authors' initial ‘manual’ analysis. The divergence is that we evidently adopted a lower threshold for determining a theme. This can be seen in the frequency of themes rank ordering (i.e., the *X* axes of Figures 4 and 5). For the *overt* self‐talking open‐ended question, *Google Notebook LM* did not pick out two of the 15 themes generated by the authors. These were in the bottom three for frequency of occurrence. For the *covert* self‐talking question, *Google Notebook LM* did not pick out seven of the 17 themes identified by the authors. These were in the bottom eight for frequency of occurrence. Furthermore, *Google Notebook LM* did tend to separate what the authors considered one theme into different themes.

## GENERAL DISCUSSION

Since socially inappropriate speaking is one of the characteristics of ADHD, we made the pre‐registered prediction that people with ADHD‐like traits would exhibit greater frequency of self‐directed speech. This proved to be the case. Responses on two different self‐directed speech inventories (STS, Experiment 1; VISQ, Experiment 2) were associated with ADHD‐like behaviours, as defined by scores on the ASRS‐v1.1. Furthermore, we found that the effect does not distinguish between overt and covert speech (Experiment 3). Interestingly, we also observed greater prevalence of self‐directed speech in ADHD‐like behaviour when participants were simply asked *How often do you talk to yourself?* (covertly and overtly), as opposed to completing a full self‐directed speech inventory (Experiment 3). This reveals how robust the association between ADHD‐like traits and talking to oneself is.

The present results necessarily lead to the question of why a greater prevalence of self‐directed speech is associated with ADHD‐like traits. Although only speculative, we present two possibilities. As with many neurodiverse conditions (e.g., ASD), individuals with ADHD are known to mask or ‘camouflage’ symptoms associated with the condition (Godfrey‐Harris & Shaw, 2023; van der Putten et al., 2024; Wicherkiewicz & Gambin, 2024). This, one can reasonably assume, is to reduce social isolation, with socially unacceptable behaviours being one of the highest causes of self‐esteem issues (Stets & Burke, 2014). Because individuals with ADHD exhibit (what is often considered to be) inappropriate talking (e.g., speaking over others, excessive talking), such behaviour can be *camouflaged* by performing the act privately. Furthermore, suppressing behaviours that feel automatic, such as emotional expression, are known to have negative consequences, for example, increased sympathetic and cardiovascular responses (Butler et al., 2003; Harris, 2001). Rather than reducing talking or not talking at all, ADHD individuals may effectively sublimate this activity by talking to oneself. Self‐directed speech could, therefore, be viewed as a coping mechanism. As Fombonne (2020) argued when referring to ASD, camouflage is essentially a strategy helping vulnerable individuals to navigate social situations. Although many adults are unaware they have ADHD (Barkley et al., 2011; Knouse et al., 2005), they will be aware of the difficulties they have with certain tasks involving, for example, organization, keeping appointments and concentrating. Such social difficulties are known to induce stress (Combs et al., 2015), hence coping mechanisms such as self‐directed speech. The fact that individuals are unlikely to consciously adopt this strategy does not negate the notion that camouflaging could be occurring. Indeed, self‐directed speech is likely to be automatic; one rarely consciously decides to speak, overtly or covertly. Furthermore, automatic camouflaging is also commonly considered to occur in ASD (Chapman et al., 2022; Cook et al., 2021; Pearson & Rose, 2021).

Relatedly, self‐directed speech may also be viewed as a displacement activity. The urge to talk (often inappropriately) could require this behaviour to be expressed in other ways. The most obvious displacement is to perform the behaviour towards oneself. This is likely to be the reason why children with no siblings use self‐directed speech more frequently than children with at least one sibling (Brinthaupt & Dove, 2012). As with socially unacceptable talking in ADHD, here, the desire to speak is effectively suppressed by the environment. The consequence is a greater prevalence of self‐directed speech. Furthermore, displacement activities most commonly occur in situations where an individual is experiencing increased levels of anxiety (e.g., Troisi, 2002), and anxiety is positively related to certain forms of self‐directed speech (i.e., *evaluative/motivational* and *presence of another's voice*; McCarthy‐Jones & Fernyhough, 2011).

These camouflaging and displacement activity hypotheses are only speculative. Indeed, no assessment of why self‐directed speech is more prevalent in people with ADHD‐like traits is one limitation of the present work. Future work could directly assess this by asking a cohort of participants with ADHD, or those with ADHD‐like traits, why they think they tend to use self‐directed speech. This could be in addition to asking participants to describe the circumstances in which they tend to self‐talk, as we did so in the present Experiment 3. Indeed, it is interesting to note that of the 17 themes generated in the open‐ended questions of that experiment (concerning *covert* self‐talk), the seventh most common response concerned the socially unacceptable nature of self‐talking. It is clear from responses that some individuals do use self‐talk as camouflage. For example, one respondent stated they self‐talk ‘when out in public and speaking out loud would be socially awkward’. Three others stated, ‘when verbalising thoughts would be rude or inappropriate’, ‘where talking out loud is questionable’ and ‘saying what I should not say in public’. These statements might provide a clue as to the *why* issue, with camouflaging being a possibility. One does, however, have to acknowledge that there may be no functional reason why self‐directed speech is more prevalent in individuals with ADHD‐like traits. The simple fact is that ADHD is associated with greater use of speech (‘excessive talking’) and speech can be directed at others, for communicating, or directed at oneself. In other words, self‐talking may just reflect greater frequency of speaking.

Finally, there has been continued interest in the attempt to determine which traits occur in both ADHD and ASD and which do not. For example, as reviewed by Taurines et al. (2012) and Craig et al. (2015), impairments in emotional recognition and executive function occur in both. In contrast, ADHD individuals are more easily distracted by emotional cues compared with ASD individuals. Similarly, the present data reveal a trait that occurs in ADHD but not in ASD. While previous work (albeit in children) has consistently shown that *spontaneous* self‐directed speech is reduced in ASD (Joseph et al., 2005; Russell et al., 1999), the present results reveal this to be the opposite for ADHD. Future work could also examine the precise scenarios in which greater self‐directed speech occurs in ADHD relative to ASD.

## AUTHOR CONTRIBUTIONS

**Ellie Benfield:** Conceptualization; writing – review and editing; software. **Geoff G. Cole:** Funding acquisition; writing – original draft; methodology; software; formal analysis; project administration; data curation; supervision.

## Supporting information


Appendix S1

## Data Availability

All raw data, means and analysis are available at https://osf.io/kdvqe/files/osfstorage.
