# Supplementary material for: Self‐directed speech and attention deficit hyperactive disorder‐like behaviours
Source: Br J Psychol. 2025 Sep 19;117(1):301–13. doi: 10.1111/bjop.70027 (PMC12783877; doi:10.1111/bjop.70027)
Supplement: Supplementary file 1 — Appendix S1 [file BJOP-117-301-s001.docx]

**An alternative way of defining ADHD-like behaviour and non-ADHD-like behaviour.**

Our initial pre-registration stated that we would define a person as having ADHD-like behaviours by performing a median split on all the ASRS scores. However, as stated in the manuscript, we updated this pre-registration to define ADHD-like behaviours using the ASRS screener (i.e., the first 6 items on the scale). Below are the results for all three experiments when ADHD-like behaviour is defined by a median split. This contrasts the way we defined ADHD in the paper, i.e., by the ASRS screener.

*Experiment 1*

After the eight participants who scored the median value (28) for ASRS scores were removed, 97 were defined as having ADHD-like behaviours and 93 were defined as not. The mean STS score for the former was 54.7 (SD = 11.3) compared with 45.5 (*SD* = 13.4) for the latter. An independent samples *t* test revealed this difference to be significant, *t*(188) = 5.14 *p* < .001, *d* = 0.7.

*Experiment 2*

After the four participants who scored the median value for ASRS scores (again, 28) were removed, 99 were defined as having ADHD-like behaviours and 95 were defined as not. The mean VISQ score for the former was 101.9 (SD = 16.4) compared with 85.7 (SD = 16.5) for the latter. An independent samples *t* test revealed this difference to be significant, *t*(192) = 6.9 *p* < .001, *d* = 0.89.

*Experiment 3*


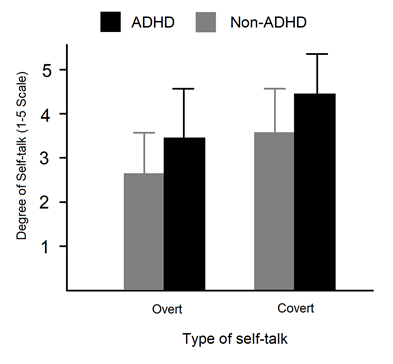
The figure below shows the mean responses on the two self-talk questions for those who reached the threshold for ADHD-like traits (i.e., above the median value; 94 participants) and those who did not (98 participants). As in the manuscript, the figure also shows standard deviations. Six participants scored the median value of 32 and were removed. A 2 x 2 mixed anova revealed a significant main effect of ADHD, *F*(1, 190) = 32.6, *p* < .001, and a significant main effect of speech type, F(1, 190) = 141.7, *p* < .001. There was however no significant interaction, *F*(1, 190) = 2.4, *p* = .12.

In sum, the analyses for Experiments 1 and 2 reveal that the basic effect (i.e., greater self-directed speech in participants with ADHD-like behaviours) is larger when median splits are used as the definition of ADHD-like traits. Results for Experiment 3 are virtually identical.

**The effect of age**

We also examined how the effects we report in each experiment are modulated by age. We undertook a median split on age to generate ‘Younger’ and ‘Older’ levels.


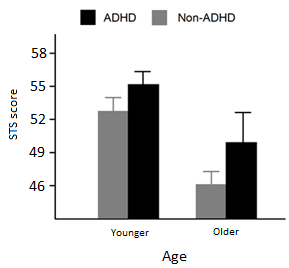
*Experiment 1*

Ten participants were omitted from this analysis because their age was the median value of 38. The adjacent figure shows the mean STS score for the younger and older participants as a function of ADHD-like traits. Standard errors are also shown. A fully independent 2 x 2 anova revealed a significant main effect of age, *F*(1, 184) = 7.6, *p* < .01, but no significant effect of ADHD, *F*(1, 184) = 2.6, *p* = .11. The interaction was not significant, *F*(1, 184) = .15, *p* = .7.

*
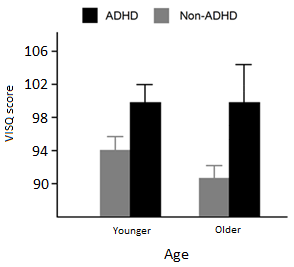
Experiment 2*

Five participants were omitted from this analysis because their age was the median value of 37. The adjacent figure shows the mean VISQ score for the younger and older participants as a function of ADHD-like traits. Standard errors are also shown. A fully independent 2 x 2 anova revealed a significant main effect of ADHD, *F*(1, 189) = 7.2, *p* < .01, but no significant effect of age, *F*(1, 189) = .3, p = .58. The interaction was not significant, *F*(1, 184) = .16, *p* = .69.

*Experiment 3*

Four participants were omitted from this analysis because their age was the median value of 43. The figure below shows the mean score for degree of self-talk for the different conditions. We performed a 2 x 2 x 2 mixed anova with age (younger, older), ADHD-like traits (ADHD, Non-ADHD), and speech type (Overt, Covert), as factors and levels. This revealed a significant main effect of age, *F*(1, 190) = 6.6, *p* = .011, a significant main effect of ADHD-like traits, *F*(1, 190) = 21.3, *p* < .001, and a significant main effect of speech type, *F*(1, 190) = 130, *p* < .001. None of the interactions were however significant; age x ADHD, *F*(1, 190) = .93, *p* = .34; age x speech type, *F*(1, 190) = 3.0, *p* = .086; a ADHD x speech type, *F*(1, 190) = .02, *p* = .9; age x ADHD x speech type, *F*(1, 190) = 1.7, *p* = .2.


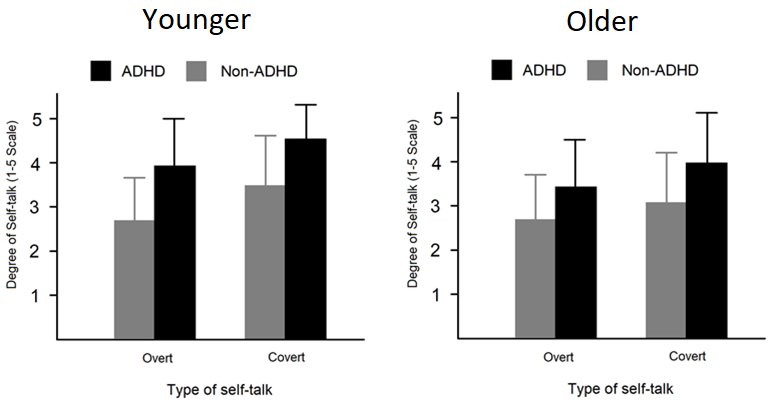


In sum, these analyses reveal that whilst overall self-talking is greater in individuals with ADHD-like traits, this effect is no larger in older relative to younger participants (or vice versa).

**The effect of gender**

We also assessed how the effects we report in each experiment are modulated by gender.


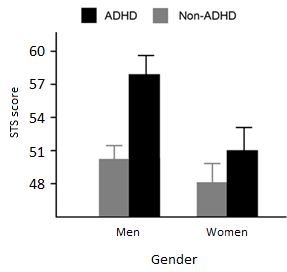
*Experiment 1*

The adjacent figure shows the mean STS score for participants who indicated their sex as man or woman as a function of ADHD-like traits. Standard errors are also shown. A fully independent 2 x 2 anova revealed a significant main effect of gender, *F*(1, 190) = 4.7, *p* = .03, and ADHD, *F*(1, 190) = 6.3, *p* = .01. The interaction was not significant, *F*(1, 190) = 1.5, *p* = .22.


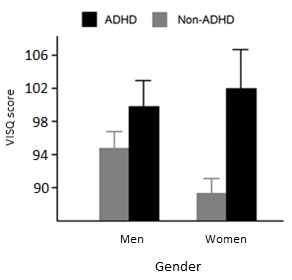
*Experiment 2*

The adjacent figure shows the mean VISQ score for the two sexes as a function of ADHD-like traits. Standard errors are also shown. A fully independent 2 x 2 anova revealed a significant main effect of ADHD, *F*(1, 188) = 10.2, *p* = .01, but not Sex, *F*(1, 188) = .55, *p* = .46. The interaction was not significant, *F*(1, 188) = 2.1, *p* = .15.

*Experiment 3*

The figure below shows the mean score for degree of self-talk for the different conditions. We performed a 2 x 2 x 2 mixed anova with gender (men, women), ADHD-like traits (ADHD, Non-ADHD), and speech type (Overt, Covert), as factors and levels. This revealed a non-significant main effect of gender, *F*(1, 194) = 2.1, *p* = .15, a significant main effect of ADHD-like traits, *F*(1, 194) = 17.4, *p* < .001, and a significant main effect of speech type, *F*(1, 194) = 117, *p* < .001. None of the interactions were however significant; gender x ADHD, *F*(1, 194) = 1.5, *p* = .22; gender x speech type, *F*(1, 194) = 2.2, *p* = .14; a ADHD x speech type, *F*(1, 194) = .4, *p* = .53; gender x ADHD x speech type, *F*(1, 194) = .07, *p* = .79.


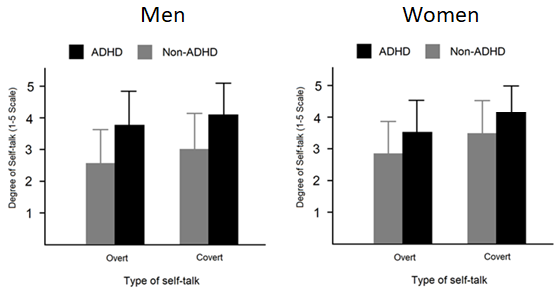


In sum, these analyses reveal that whilst overall self-talking is greater in individuals with ADHD-like traits, this effect is no larger in men relative to women participants (or vice versa).

**Thematic analysis performed by *Google Notebook LM.***

In two separate analyses, we copied all participant responses from the two open-ended questions of Experiment 3 and pasted them into *Google Notebook LM (*via a pdf document as required by the system). We performed some very minor editing on the raw participant responses for this analysis. Here, we deleted responses in which a participant stated something akin to, “I don’t do this” (i.e., self-talking), and also corrected an odd text coding issue (e.g., “I‚Äôm” replaced with “I’m”). Furthermore, on some occasions, participants answered the second of the two questions with the phrase “As above”, “As just stated”, or “Same as before”. Here, we pasted their response from the first question into the second.

For the first of the two open-ended questions, we then asked the system to “Pick out the 15 most common themes”; 15 because the authors’ (non-AI) analysis presented in the paper had generated 15 themes (as shown on the *X* axis of Figure 4). For the second of the two open-ended questions, we asked the system to “Pick out the 17 most common themes”; 17 because the authors’ analysis had generated 17 themes (as shown on the *X* axis of Figure 5).

Below is the raw text produced by *Google Notebook LM* for the two questions. Immediately after each of the themes we present the equivalent theme generated by the authors (i.e., those pertaining to those on the *X* axes of figures 4 and 5).

Note that we (the authors) think its reference to “our conversation history” concerns one previous question we also asked about the same two documents. This was “Perform a thematic analysis on the source”.

***First open-ended question: “In about 2-3 sentences, can you describe the circumstances in which you talk to yourself out loud?”***

***The Google Notebook LM text:***

Based on the provided sources and our conversation history, here are the 15 most common themes regarding why and when individuals engage in self-talk:

1. Problem-Solving & Working Through Solutions: Individuals frequently use self-talk to address challenges, work out calculations (e.g., maths or adding up), resolve problems, work through solutions, and figure something out. This includes clarifying complex scenarios or memos, and finding "the right answers". ***“Coming up with a solution“.***

2. Planning & Organising Tasks Logically: Self-talk is a crucial tool for mental organisation and task execution. People talk to themselves to plan projects step-by-step, organise daily tasks, make lists, remind themselves what to do, and ensure they are doing things in a logical way without missing steps. This occurs at work, at home, and for general errands. ***“Plans/Organising“.***

3. Self-Motivation & Encouragement: A prominent theme is using self-talk for personal motivation and encouragement. This includes giving oneself a pep talk for a presentation or meeting, firing oneself up for sports, building confidence, or simply to urge oneself to "keep going" during difficult or undesirable tasks. ***“Encouragement/Motivation“.***

4. Emotional Regulation & Calming: Self-talk is widely used to manage emotions and achieve a state of calm. This is particularly evident when individuals are anxious, stressed, worried, upset, or panicking. It helps to "remain calm" or "calm myself down". ***“Thinking/Handling emotions“.***

5. Memory Aid & Reminders: Individuals engage in self-talk to remember what to do, recall misplaced items, or reinforce instructions from paper. It helps to ensure tasks are not forgotten. ***“Remembering“.***

6. Dealing with Mistakes & Self-Reprimand: A common reason for self-talk is to acknowledge and react to one's own errors or poor performance. This includes telling oneself off, expressing frustration for "letting myself down", "swearing at myself", or noting when one has "done something stupid". ***“Discipline“.***

7. Concentration & Focus Enhancement: Self-talk aids in maintaining focus and concentration, especially during tasks that require significant mental effort. It can involve talking through a safety-critical role step-by-step, or simply to "try to concentrate on a task". ***“Concentrating“.***

8. Rehearsal & Social Navigation (including self-censorship): Individuals use self-talk to mentally prepare for social interactions and prevent inappropriate verbalisations. This includes rehearsing conversations, practising work situations, or public speaking, and stopping oneself from saying something inappropriate. ***“Practicing a conversation/argument“.***

9. Reflection & Processing Past Events: Self-talk is employed to rethink or replay past situations, make sense of things that have bothered them, or process problems with an emotional component. It allows individuals to "relive a situation I could have resolved differently" or process "something which has happened to me". ***“Thinking/Handling emotions“.***

10. Managing Frustration, Annoyance & Anger: Distinct from general calming, this theme highlights self-talk as a response to feelings of irritation, exasperation, or anger. It occurs when things go wrong, items are lost, or when "people annoy me". ***“Thinking/Handling emotions“.***

11. Coping with Boredom & Seeking Mental Stimulation: Self-talk often fills voids, serving as a mental activity when individuals are bored, doing "mindless" activities, or simply "just talking myself through what I'm doing". ***Possibly incorporates what the authors’ coded as “Driving”.***

12. Expressing Heightened Emotions & Exclamations: Self-talk can be a spontaneous output of intense feelings. This includes verbalising "absolutely everything" when "overly happy/excited", exclaiming "silly me" or "right", or simply expressing how one feels about an action or achievement. ***“Exclamation“.***

13. Aid During Physical Tasks/Activities: Self-talk frequently accompanies various activities, acting as a running commentary or procedural aid. This includes housework, shopping, driving, walking, or sports. ***“Doing task and Sports“.***

14. When Alone or in Private Settings: A very common contextual trigger for self-talk, particularly vocalised self-talk, is when individuals are by themselves. This occurs at home, in the car, in their room, or in other private environments. ***“Alone/In private“.***

15. Habitual & Unconscious Dialogue: For many, self-talk is not just a tool but a constant, pervasive, and often unconscious aspect of their daily experience. It occurs "all day in every scenario", "every day", "without me realising it whilst I'm doing something", and can be "just random" or "in all situations". For one individual, it is even described as "All part of my Aspergers!", implying an integrated cognitive style. **[This theme was not generated by the authors].**

**END OF GOOGLE NOTEBOOK LM TEXT.**

There were two themes picked out by the authors but not by *Google Notebook LM*. These were ***Inner strength*** and **Singing.** The first of these was considered as ***Motivation*** by the authors.

***Second open-ended question: “In about 2-3 sentences, can you describe the circumstances in which you talk to yourself in your head?”***

***The Google Notebook LM text:***

Drawing on the information in the provided sources and our conversation history, a thematic analysis reveals 17 common themes regarding why and when individuals engage in self-talk:

1. Problem-Solving & Working Through Solutions: Individuals frequently use self-talk to address challenges, work out calculations, resolve problems, figure something out, and find "the right answers" [Implied by "thinking things through" 2]. This includes clarifying complex scenarios or memos. ***“Thinking/monologue”****.*

2. Planning & Organising Tasks Logically: Self-talk is a crucial tool for mental organisation and task execution. People talk to themselves to plan projects step-by-step, organise daily tasks, make lists, remind themselves what to do, and ensure they are doing things in a logical way without missing steps. This occurs at work, at home, and for general errands. ***“Thinking through tasks/Making lists“****.*

3. Self-Motivation & Encouragement: A prominent theme is using self-talk for personal motivation and encouragement. This includes giving oneself a pep talk, firing oneself up for a presentation or meeting, building confidence, or simply to urge oneself to "keep going" during difficult or undesirable tasks. ***“Motivation/Encouragement“ and “Inner strength”.***

4. Emotional Regulation & Calming: Self-talk is widely used to manage emotions and achieve a state of calm. This is particularly evident when individuals are anxious, stressed, worried, or upset. It helps to "remain calm" or "calm myself down". ***“Handling emotions“.***

5. Memory Aid & Reminders: Individuals engage in self-talk to remember what to do, recall misplaced items, or reinforce instructions. It helps to ensure tasks are not forgotten. ***“Remembering“.***

6. Dealing with Mistakes & Self-Reprimand: A common reason for self-talk is to acknowledge and react to one's own errors or poor performance. This includes telling oneself off, expressing frustration for "letting myself down", "swearing at myself", or noting when one has "done something stupid". ***“Discipline“.***

7. Concentration & Focus Enhancement: Self-talk aids in maintaining focus and concentration, especially during tasks that require significant mental effort. It can involve talking through a safety-critical role step-by-step, or simply to "try to concentrate on a task". ***“Focusing“.***

8. Rehearsal & Preparation for Social Interaction: Individuals use self-talk to mentally prepare for social interactions. This includes rehearsing conversations, practising work situations, and preparing for public speaking. It helps individuals process how statements sound and navigate potential social encounters. ***“Thinking through conversations/scenarios“.***

9. Social Censorship & Preventing Inappropriate Outbursts: A very common use of self-talk is to prevent inappropriate verbalisations. It acts as a social filter, stopping individuals from "saying something inappropriate" or "rude" when annoyed with people or when having strong opinions about others. ***“Not socially acceptable out loud“.***

10. Reflection & Processing Past Events: Self-talk is employed to rethink or replay past situations, make sense of things that have bothered them, or process mistakes. It allows individuals to "relive a situation I could have resolved differently" or process "something which has happened to me". ***“Handling emotions“.***

11. Managing Frustration, Annoyance & Anger: Distinct from general calming, this theme highlights self-talk as a response to feelings of irritation, exasperation, or anger. It occurs when things go wrong, items are lost, or when "people annoy me". ***“Handling emotions“.***

12. Coping with Boredom & Seeking Mental Stimulation: Self-talk often fills voids, serving as a mental activity when individuals are bored, doing "mindless" activities, or simply "just talking myself through what I'm doing". It can also be because they "Just like the sound of my own voice in my head". **[This theme was not generated by the authors].**

13. Expressing Heightened Emotions & Exclamations: Self-talk can be a spontaneous output of intense feelings. This includes verbalising "absolutely everything" when "overly happy/excited", exclaiming "silly me" or "right", or simply expressing how one feels about an action or achievement. ***“Handling emotions“.***

14. Aid During Physical Tasks/Activities: Self-talk frequently accompanies various activities, acting as a running commentary or procedural aid. This includes housework, shopping, driving, walking, sports, and work-related tasks. ***“Thinking/Doing activity/Sports“.***

15. When Alone or in Private Settings: A very common contextual trigger for self-talk, particularly vocalised self-talk, is when individuals are by themselves. This occurs at home, in the car, in their room, in the bath, or during specific tasks when alone. **[This theme was not generated by the authors].**

16. Habitual & Unconscious Dialogue: For many, self-talk is not just a tool but a constant, pervasive, and often unconscious aspect of their daily experience. It occurs "all day in every scenario", "every day", "without me realising it whilst I'm doing something", and can be "just random" or "in all situations". **[This theme was not generated by the authors].**

17. Self-Understanding & Processing Current Situations: Self-talk is used for internal clarification and making sense of one's immediate reality or internal state. It helps individuals "understand or process what is happening", or "rationalize something", especially when overwhelmed by thoughts. ***“Handling emotions“.***

**END OF GOOGLE NOTEBOOK LM TEXT.**

There were seven themes picked out by the authors but not by *Google Notebook LM*. These were ***“During conversations/In social setting“*, *“Overthinking“*, *“Writing“*, *“Imaginary world“*, *“Reading“, “Songs“,* and *“Visualize“*.**

As noted in the article, the AI thematic analysis shows good consistency with the authors’ ‘manual’ analysis. The divergence is that the authors appeared to have a lower threshold for determining a theme. For the *overt* self-talking question, *Google Notebook LM* did not pick out two generated by the authors. These two were in the bottom three for frequency of occurrence (see Figure 4 of the article). For the *covert* self-talking question, *Google Notebook LM* did not pick out seven. These were in the bottom eight for frequency of occurrence (see Figure 5 of the article). AI did however tend to separate what the authors considered as one theme into different themes.
